# Pathology and Epidemiology of Ceruminous Gland Tumors among Endangered Santa Catalina Island Foxes (*Urocyon littoralis catalinae*) in the Channel Islands, USA

**DOI:** 10.1371/journal.pone.0143211

**Published:** 2015-11-30

**Authors:** T. Winston Vickers, Deana L. Clifford, David K. Garcelon, Julie L. King, Calvin L. Duncan, Patricia M. Gaffney, Walter M. Boyce

**Affiliations:** 1 Institute for Wildlife Studies, Arcata, California, United States of America; 2 Karen C. Drayer Wildlife Health Center, School of Veterinary Medicine, University of California Davis, Davis, California, United States of America; 3 Wildlife Investigations Lab, California Department of Fish and Wildlife, Rancho Cordova, California, United States of America; 4 Catalina Island Conservancy, Avalon, California, United States of America; 5 Department of Pathology, Microbiology, and Immunology, School of Veterinary Medicine, University of California Davis, Davis, California, United States of America; 6 Departments of Pathology and Medicine, University of California San Diego, San Diego, California, United States of America; Colorado State University, UNITED STATES

## Abstract

In this study, we examined the prevalence, pathology, and epidemiology of tumors in free-ranging island foxes occurring on three islands in the California Channel Islands, USA. We found a remarkably high prevalence of ceruminous gland tumors in endangered foxes (*Urocyon littoralis catalinae*) occurring on Santa Catalina Island (SCA)—48.9% of the dead foxes examined from 2001–2008 had tumors in their ears, and tumors were found in 52.2% of randomly-selected mature (≥ 4 years) foxes captured in 2007–2008, representing one of the highest prevalences of tumors ever documented in a wildlife population. In contrast, no tumors were detected in foxes from San Nicolas Island or San Clemente Island, although ear mites (*Otodectes cynotis)*, a predisposing factor for ceruminous gland tumors in dogs and cats, were highly prevalent on all three islands. On SCA, otitis externa secondary to ear mite infection was highly correlated with ceruminous gland hyperplasia (CGH), and tumors were significantly associated with the severity of CGH, ceruminous gland dysplasia, and age group (older foxes). We propose a conceptual model for the formation of ceruminous gland tumors in foxes on SCA that is based on persistent, ubiquitous infection with ear mites, and an innate, over exuberant inflammatory and hyperplastic response of SCA foxes to these mites. Foxes on SCA are now opportunistically treated with acaricides in an attempt to reduce mite infections and the morbidity and mortality associated with this highly prevalent tumor.

## Introduction

**Fig 1:**
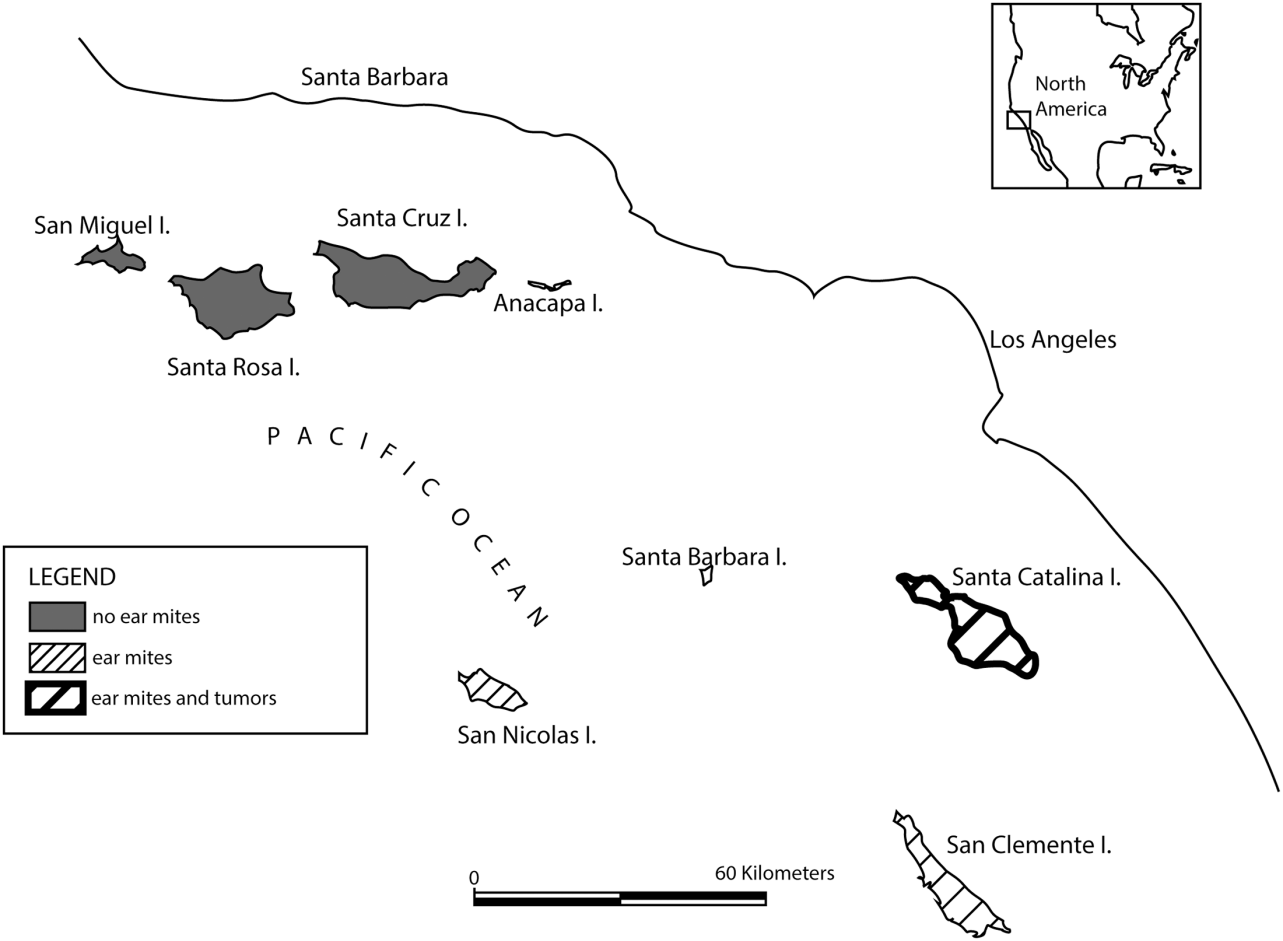
Map of the Channel Islands (California, USA). Distribution of foxes that are uninfected or infected with ear mites, and foxes that have ceruminous gland tumors. Island foxes do not reside on Santa Barbara or Anacapa islands.

Six subspecies of island fox (*Urocyon littoralis)*, diminutive descendants of the mainland gray fox (*U*. *cinereoargenteus)*, are found on the Channel Islands located off the mainland coast of southern California, USA [1] (Fig 1). Northern island subspecies include the San Miguel Island (SMI) Fox (*U*. *l*. *littoralis)*, Santa Rosa Island (SRI) Fox (*U*. *l*. *santarosae)*, and Santa Cruz Island (SCZ) Fox (*U*. *l*. *santacruzae)*, and southern island subspecies include Santa Catalina Island (SCA) Fox (*U*. *l*. *catalinae)*, San Nicolas Island (SNI) Fox (*U*. *l*. *dickey)*, and San Clemente Island (SCI) Fox (*U*. *l*. *clementae)*. Predation, disease, and other factors led to catastrophic declines in fox numbers to < 1,500 for all six subspecies combined in 2002 [1,2], and all northern island subspecies (SMI, SRI, SCZ), and one southern island subspecies (SCA), were listed as endangered in 2004 [3]. Population recovery of all 4 federally endangered subspecies is occurring, but no subspecies have been delisted or down-listed to date [1].

The only terrestrial mammals native to the California Channel islands include island fox, deer mice (*Peromyscus maniculatus*), island spotted skunk (*Spilogale gracilis amphiala*, SCZ and SRI only), the Catalina California ground squirrel (*Otospermophilus beecheyi nesioticus*; SCA only), Western harvest mouse (*Reithrodontomys megalotiss*; SCZ, SCA, SCI), and the Santa Catalina Island shrew (*Sorex ornatus willeti*; SCA only) [4]. However, many non-native terrestrial mammals were introduced accidentally or intentionally onto one or more of the islands, including domestic cats (*Felis catus*) and dogs (*Canis domesticus*), raccoons (*Procyon lotor*), feral pigs (*Sus scrofa*), elk (*Cervus canadensis*), mule deer (*Odocoileus hemionus*), wild turkeys (*Meleagris gallopavo*), bison (*Bison bison*), domestic sheep (*Ovis aries*), and domestic goats (*Capra hircus*) [4]. These introduced species severely perturbed the island ecosystems, altering the landscape [5] and provided a potential conduit for parasites and pathogens from the mainland to reach the islands. The threat posed by introduced novel pathogens has been demonstrated repeatedly around the globe [6,7], and a non-native species (raccoons) almost certainly introduced canine distemper virus (CDV) into the SCA island fox population in the 1990’s causing an epidemic that reduced fox numbers by 95% [8].

In 2001, ceruminous gland tumors (carcinomas and adenomas) were detected in the ears of several foxes on SCA [8,9] and nearly all of the foxes that were opportunistically examined were found to be infected with the common ear mite of dogs and cats (*Otodectes cynotis*). Ear mites also appeared to be common on two other islands (SNI, SCI), but opportunistic sampling suggested that tumors only occurred in SCA foxes. Subsequent investigations suggested that tumors might be common in mature foxes. These discoveries raised concerns about the etiology, prevalence and impact of cancer on a fragile recovering population, and prompted this systematic investigation and our follow-up companion study [10].

In this paper, we provide the first description of the pathogenesis of ear lesions and ceruminous gland tumors in island foxes, and evaluate the prevalence and epidemiology of tumors among foxes on the three islands where ear mites are prevalent (SCA, SCI, SNI). We identify risk factors for tumor development, and propose a mechanism whereby ear mite infection incites a chronic inflammatory response leading to tumor development only in SCA foxes. In the companion paper by Moriarty et al. [10], we present the results of a field trial on Santa Catalina Island where we tested this hypothesis by comparing ear pathology in two groups of foxes—one group treated for ear mites and one untreated comparison group.

## Materials and Methods

### Ethics statement

Animal capture and biological sampling of SCA, SNI, and SCI foxes was authorized under a U.S. Fish and Wildlife Service (USFWS) Federal 10(a)1(A) permit (No. TE 744878–9) and a Memorandum of Understanding with the California Department of Fish and Wildlife (CDFW). All procedures were reviewed and approved by the University of California, Davis, Institutional Animal Care and Use Committee (Protocol No. 12248).

### Study area

The study was conducted on the three southern California Channel Islands (SCA, SNI, SCI) that support island fox populations (Fig 1), since ear tumors and ear mite infections have never been observed among foxes on the northern Channel islands (SCZ, SMI, SRA) [9]. Santa Catalina Island has an area of 194 km^2^ and is located 32 km southwest of Long Beach, California (33° 24’ N, 118° 24’ W). Approximately 88% of the island is managed by the Catalina Island Conservancy, and the remainder is privately owned. Over 4000 human residents live on SCA year-round, and approximately 800,000 people visit the island each year. The SCA fox population dropped to about 157 animals in 2000, and then increased to about 1115 animals by 2012 [1]. San Clemente Island (32° 47’ N, 118° 25’ W) has an area of 145 km^2^ and is owned and managed by the U.S. Navy. The SCI fox population fell to less than 500 adults in 2000, and increased to approximately 800 adults by 2012 [11]. San Nicolas Island (33° 13’ N, 119° 26’ W) covers an area of 59 km^2^, is also owned and managed by the U.S. Navy. In contrast to the other islands, the fox population on SNI remained relatively stable from 2000 to 2011 with the adult population fluctuating around 500 animals [12]. Residents and visitors on SCI and SNI are primarily navy personnel, contractors, and researchers.

### Study animals and sampling

Archived aural tissues suitable for pathology studies were obtained from dead foxes found on the islands from 2001–2008 (SCA, n = 47; SCI n = 50; SNI, n = 50). In 2007–2008, we conducted physical examinations of randomly selected live-captured adult (>1 yr) foxes on SCA (n = 105), SCI (n = 59), and SNI (n = 89), and collected aural biopsies from a subset of these animals (SCA = 64, SCI = 20, SNI = 22) as well as additional SCA foxes examined separately (n = 47). Field capture was accomplished via box traps (Tomahawk Live Trap Company, Tomahawk, Wisconsin, USA) that were set and baited with a combination of dog kibble, canned cat food, and berry paste. Traps were placed along transects or on grids that incorporated all accessible major habitat types and geographic regions of the 3 islands. All traps were checked each morning, and foxes were examined, sampled, and released at the site of capture. Foxes were blindfolded and manually restrained, and physical and otoscopic examinations conducted. All examinations and sampling were conducted by the same veterinarian (TWV), and baseline data collected included passive integrated transponder (PIT) tag number, sex, age, weight, physical condition, and ear canal observations. Adult foxes 1–3 years old were classified as “young” and foxes ≥ 4 years old were classified as “mature.” No foxes less than 1 year of age were sampled. If exact age could not be determined from previous capture history, foxes were assigned to age groups using tooth wear patterns [13,14] and foxes were categorized as “young” if they had Class 1 dentin exposure patterns or “mature” if they had Class 2–4 dentin exposure patterns.

An initial otoscopic exam was performed to detect and quantify ear mite infections, and results were scored from 0–4 based on the intensity of infection observed. Two sterile swabs were inserted into each ear to obtain samples for bacterial and fungal/yeast culture, respectively. A separate sterile cotton swab was then used to collect mites from the affected ear(s) and placed in 70% ETOH for later analyses. The ear canals were then cleaned with additional cotton swabs, and a more detailed otoscopic examination performed to detect any visible abnormalities. All foxes with visible tissue masses [gross proliferative lesions (GPL)] in their ear canals had biopsies taken of the GPL. Topical and subdermal lidocaine were applied in the area for local anesthesia, then a 3–6 mm tissue sample was taken from the GPL with a sterile skin biopsy punch or biopsy forcep, and fixed in 10% buffered formalin for histopathology. A second biopsy sample was taken adjacent to the first and frozen for future molecular diagnostics. In cases where large numbers of individual masses were present in a fox’s ear, the largest was sampled. Foxes without GPL from each island were also chosen by systematic random selection for biopsy. Samples from unaffected foxes were taken from the caudolateral aspect of the lower vertical ear canal where GPL commonly occurred in affected foxes.

### Pathology

Necropsy samples from dead foxes and biopsy samples from live foxes were evaluated by the same pathologist who was blinded to the island of origin and all other data associated with each individual fox. Tumors were identified and classified as ceruminous gland adenomas (CGA) or ceruminous gland carcinomas (CGC—including subtypes termed ceruminous gland adenocarcinoma and cystadenocarcinoma), and histopathologic changes thought to precede or be associated with the development of cancerous and non-cancerous tumors were noted (Table 1). A scoring system was used to quantify the degree of otitis externa and ceruminous gland hyperplasia detected on histopathologic evaluation (Table 2). The prevalence of otitis, CGH, and tumors (CGA and CGC) in the SCA, SNI, and SCI fox populations from 2001–2008 was determined from examination of dead foxes. The prevalence of ear mites, GPL, otitis, CGH, and tumors (CGA and CGC) in live foxes was estimated from adults captured, examined, and sampled in 2007–2008.

**Table 1:** Pathology terms and definitions.

Adenoma—a benign tumor of glandular epithelial cells; adenomas may be space occupying but epithelial cells do not breach the glands basement membrane.
Adenocarcinoma and cystadenocarcinoma—a malignant tumor of glandular epithelial cells, often with a glandular or gland-like pattern but may be composed of solid sheets.
Cancer—a malignant tumor in which normal cells transform into tumor cells with uncontrolled (or unregulated) cell growth
Carcinoma—a malignant tumor of epithelial cells
Ceruminous gland—specialized sweat glands in the subcutaneous tissues of the external auditory canal that produce cerumen (ear wax)
Dysplasia—when comparing to neoplasia, dysplasia is abnormal or disorganized growth of cells, often of immature cells, and can be an early indicator of a neoplastic transformation
Ectasia—a non-cancerous dilation, distension or expansion of a glandular or ductular lumen [tumor can have areas that are ectatic too]
Hyperplasia—an increase in the number of cells in a tissue; a normal physiologic response that is reversible (as opposed to abnormal cell growth and division in dysplasia and neoplasia)
Gross proliferative lesion—a lesion or mass that can be detected with the naked eye or by palpation
Otitis externa—inflammation of the external ear canal
Tumor—also called neoplasm, is an abnormal enlargement of tissue that occurs due to uncontrolled cell growth; may be benign (non-cancerous) or malignant (cancer) [Note—the basic definition of tumor is a swelling or enlargement that could be inflammation or hyperplasia or neoplasia. The most common use of course is that tumor = neoplasia]

**Table 2:** Scoring criteria: otoscopic assessment of mites and histopathologic assessment of otitis and ceruminous gland hyperplasia (CGH).

Mite score	
0	No mites
1	Few mites, scattered mites throughout the ear canal
2	One cluster of mites, some scattered mites throughout the ear canal
3	Two clusters of mites, some scattered mites throughout the ear canal
4	Multiple clusters of mites throughout the ear canal
Otitis score	
0	None
1	Few plasma cells, mast cells, neutrophils or eosinophils in the dermis
2	Moderate numbers of plasma cells, lymphocytes, mast cells, neutrophils or eosinophils present in the epidermis and/or expanding the dermis
3	Abundant plasma cells, lymphocytes, mast cells, neutrophils or eosinophils in the epidermis and dermis with destruction of epidermal and dermal components
CGH score	
0	None
1	Mild gland ectasia and piling up of cells within glands
2	Moderate gland ectasia and increased number of glands
3	Marked gland ectasia and adenomatous gland clusters

### Mites, microbes, viruses, and toxins

Because differences among ear mites on the different islands might play a role in tumor development, we conducted morphologic analyses of mites from foxes on SCA, SNI, and SCI. Nine morphologic characteristics were measured on 30 male and 30 female mites collected from foxes on each island as previously described [15]. These characteristics included body length, body width posterior, body width anterior, outer opisthosomal setae length, knob length medial, knob length lateral, knob length difference, knob width basal, and knob width distal. Similar measurements were performed on mites collected from domestic cats on SCA and mainland California, and discriminant function analysis [15,16] was used to analyze similarities and differences among mites from these five sample groups (SCA fox, SNI fox, SCI fox, SCA cat, mainland cat). We also attempted to generate and analyze DNA sequence data from the internal transcribed spacer region [17], but were unable to obtain usable sequence data from individual mites.

Ear swabs from a subset of live foxes whose ears were biopsied on SCA (n = 108), SCI (n = 20), and SNI (n = 22) were cultured to assess whether bacterial or fungal pathogens differed between foxes with and without otitis and tumors. Samples were cultured at a commercial laboratory (IDEXX Laboratories, West Sacramento, CA) for aerobic and anaerobic bacteria, yeast, and fungi using standard microbiological methods. Organisms were identified to the genus and (when possible) species level. Because herpesviruses and papillomaviruses have been associated with tumors [18–20], nested herpesvirus primers were utilized to amplify a partial sequence of the DNA-dependent-DNA polymerase gene [21], and degenerate papillomavirus primers were utilized to amplify a partial sequence of the E1 protein [22]. Tissue infected with macropodid-herpesvirus 3 and a canine oral papilloma were used as positive controls.

Because toxic chemicals have been associated with tumor development in other species [23–25], we evaluated exposure to polychlorinated biphenyls (PCBs) and organochlorines (OCs). Sera from foxes on SCA (n = 10, 5 with and 5 without cancer), SCI (n = 5), and SNI (n = 5) were tested for PCBs and OCs at the California Animal Health and Food Safety Laboratory in Davis, California, USA. Adipose tissue extracts from recently deceased foxes (five foxes from each of the three islands) were also screened for OC pesticides and total PCBs by gas chromatography with electron capture detection. If OCs or PCBs were detected, their presence was confirmed using gas chromatography—mass spectrometry. The OC pesticides included in the screen were hexachlorobenzene, chlordane, endosulfan I and II, endrin, DDT (o,p’ and p,p’), DDE (o,p’ and p,p’), DDD (o,p’ and p,p’), methoxychlor, lindane, hepatachlor, hepatachlor expoxide, aldrin, dieldrin, dicofol, mirex, toxaphene, and BHC.

### Tumor prevalence and risk factor analysis

Two of our primary goals were to: 1) estimate the prevalence of tumors in the SCA, SCI, and SNI fox populations; and 2) identify those factors associated with the presence of ear lesions and tumors. For prevalence estimation we utilized results of random examinations of foxes trapped on all 3 islands (SCA = 105, SCI = 59, SNI = 89) with aural biopsies of those observed to have GPL in one or both ears. For risk factor analyses, data from all biopsied live-sampled foxes (SCA = 111, SCI = 20, SNI = 22) were included and adenoma and carcinoma cases were combined into a single case group (tumors) on the assumption that similar risk factors would lead to either neoplastic condition. Chi-square tests were used to determine if tumor cases were associated with age group, sex, presence of dysplasia, presence of yeast, and presence of bacteria. Associations between tumors and ceruminous gland hyperplasia score, otitis severity score, mite intensity score, the number of pathogenic microbial species cultured, and the total number of microbial species cultured were evaluated using the Kruskal-Wallis one way analysis of variance by ranks. To investigate the differences in risk factors between islands, regardless of tumor status, the same set of risk factors above were compared among SCA, SCI, and SNI foxes. If overall Kruskal-Wallis test results were significant, pairwise comparisons to determine which islands differed were made using Mann-Whitney U tests with an adjusted p-value (Holms sequential Bonferroni adjustment). Spearman’s rank was used to determine whether scores for otitis severity, CGH severity and mite intensity were correlated.

On SCA, a multiple logistic regression model was used to evaluate associations between tumor presence in live-sampled foxes and multiple risk factors (CGH, age, sex, dysplasia, mites, otitis) simultaneously. Variables were selected for inclusion in the model based on the univariate analyses and likelihood ratio (LR) tests while overall model fit was assessed using the Hosmer-Lemeshow test statistic [26]. The strengths of associations were estimated using logistic odds ratios (OR) and 95% binomial confidence intervals [27]. Analyses were performed using STATA ver 11 (STATA corp, College Station, TX, USA).

## Results

### Pathology

Gross proliferative lesions provided the first outwardly visible indication that pathologic changes were occurring in the ear, and GPL were detected in 43.8% of all randomly examined live SCA foxes. Among mature SCA foxes, 76.1% had GPL, and 70.6% of those GPL were classified as tumors upon histopathologic analysis. Although GPL were detected in 20% of SCI foxes and 32% of SNI foxes, none of these lesions were classified as tumors based on histologic exam. Non-tumor causes of GPL included florid ceruminous gland hyperplasia, healed cartilage fractures, and epidermal hyperplasia. No tumors were detected in any GPL-negative foxes [n = 19 (SCA), n = 16 (SCI), n = 15 (SNI)].

**Fig 2:**
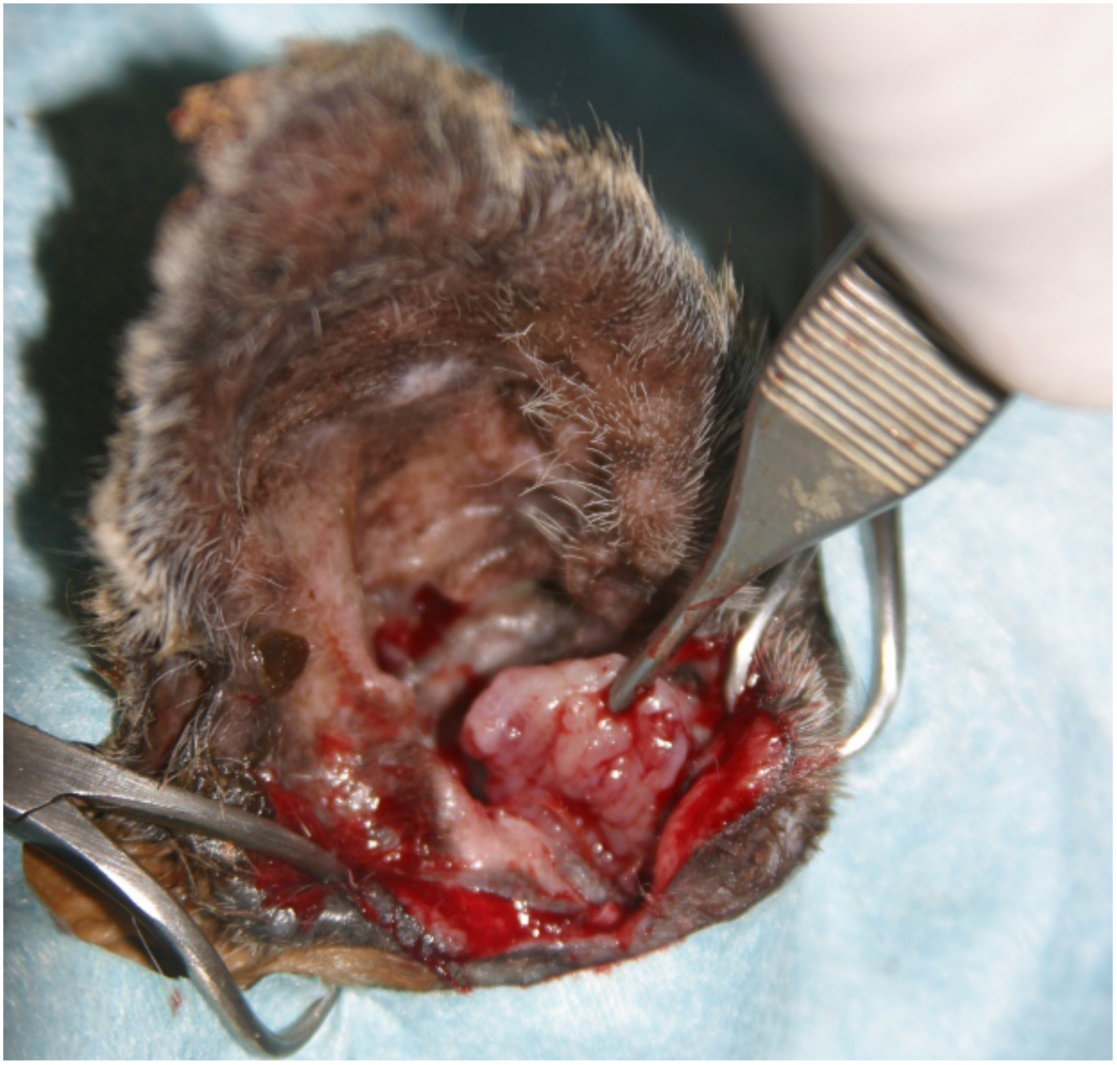
Photo of ceruminous gland tumor within ear canal of a Santa Catalina Island fox.

**Fig 3:**
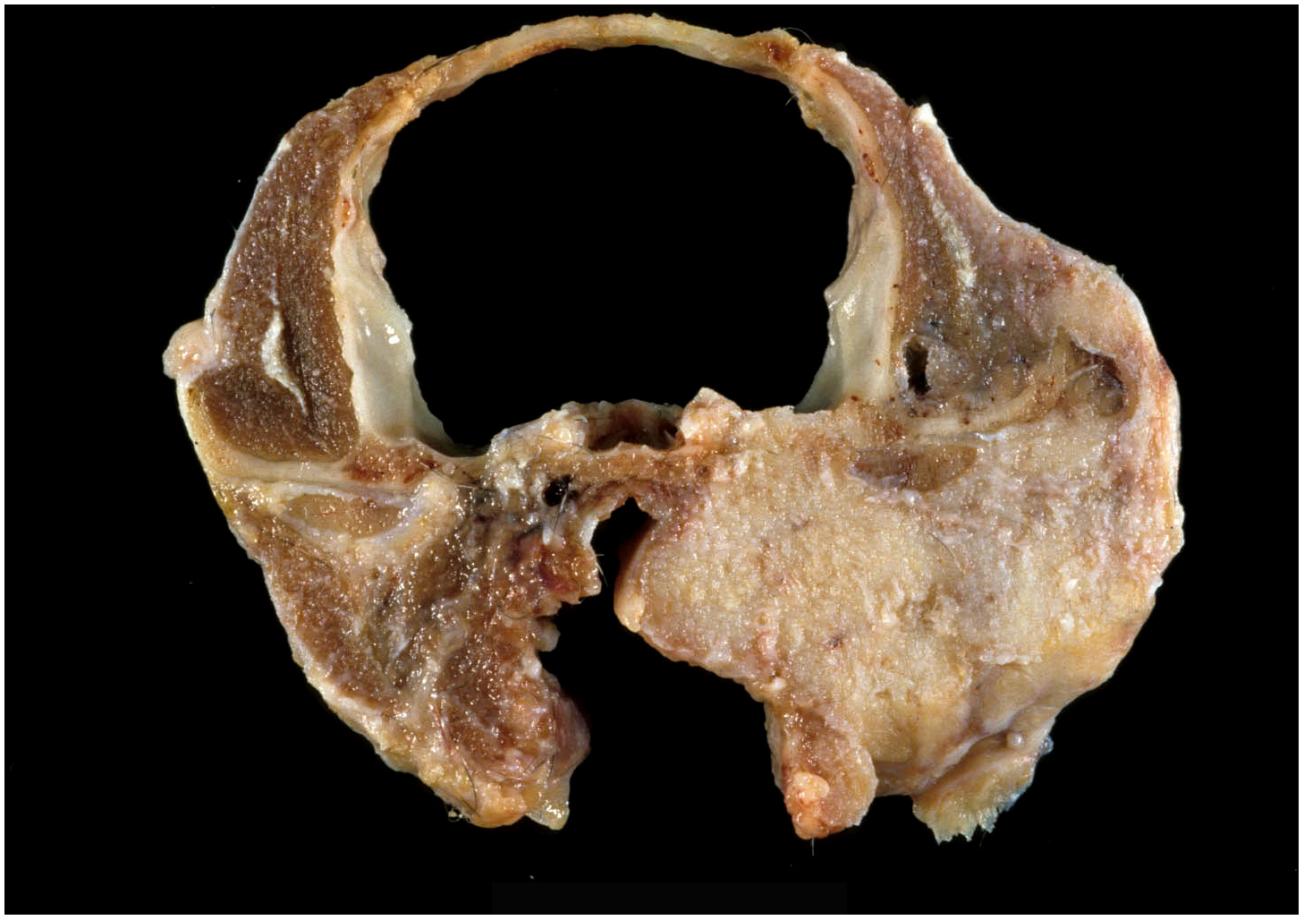
Photo of ceruminous gland tumor after local invasion into adjacent bulla tympanica, pharynx, temporal, and occipital bones and skull of a Santa Catalina Island fox.

**Fig 4:**
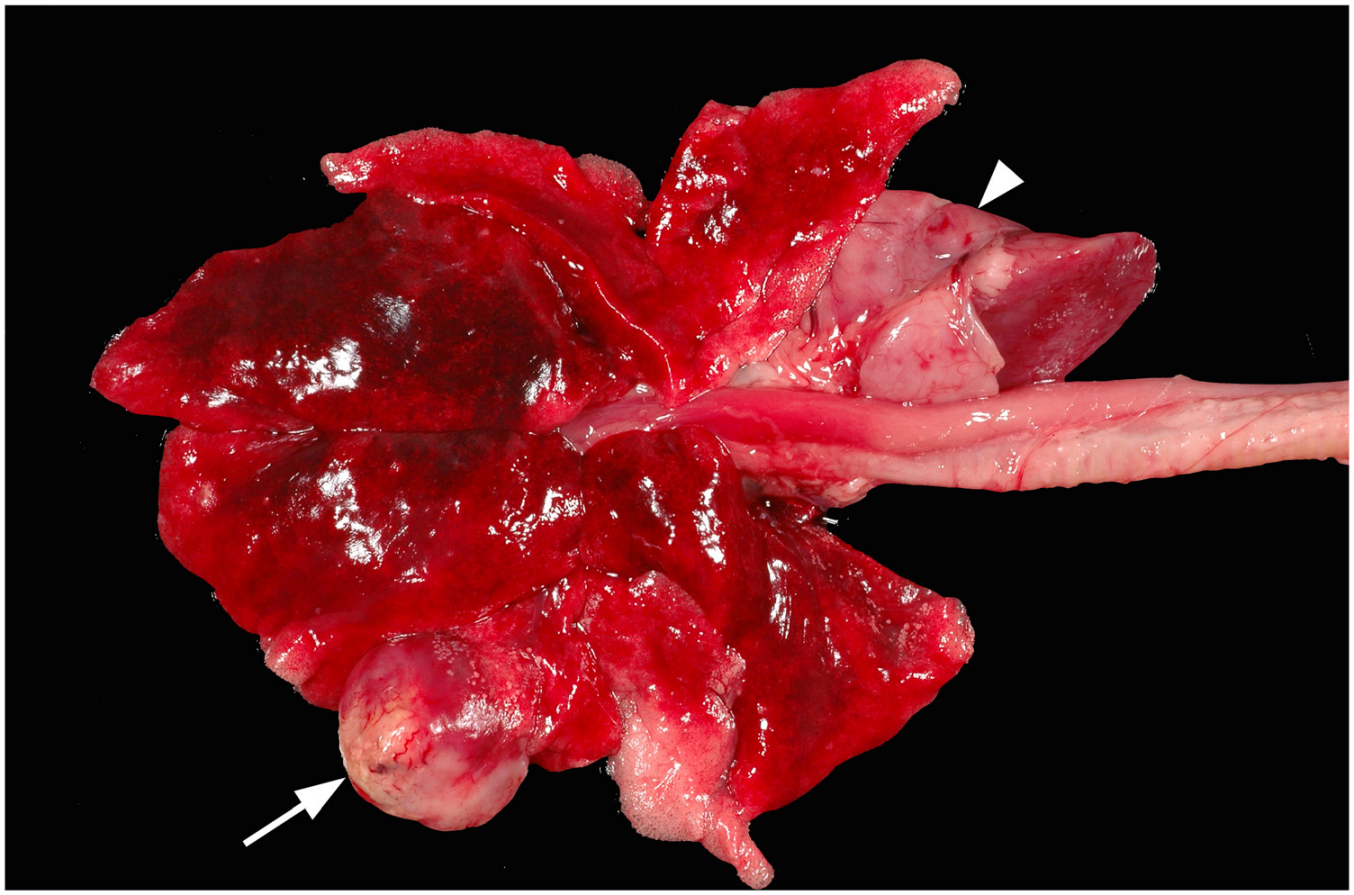
Photo of ceruminous gland tumor after metastasis to lung, forming discrete tumors (arrow) and infiltrating pulmonary parenchyma (arrowhead) of a Santa Catalina Island fox.

On gross examination, ceruminous gland tumors varied in size, shape, and invasiveness. Adenomas were often discrete, raised, round masses with a smooth surface. Carcinomas had irregular borders and could be exophytic and pedunculated with an ulcerated surface. Some tumors occluded the entire ear canal and extended beyond the external pinna (Fig 2). Less frequently, carcinomas were locally invasive, infiltrating adjacent bone and in one case extending into the skull (Fig 3), or exhibiting metastasis to regional lymph nodes and lung (Fig 4).

**Fig 5:**
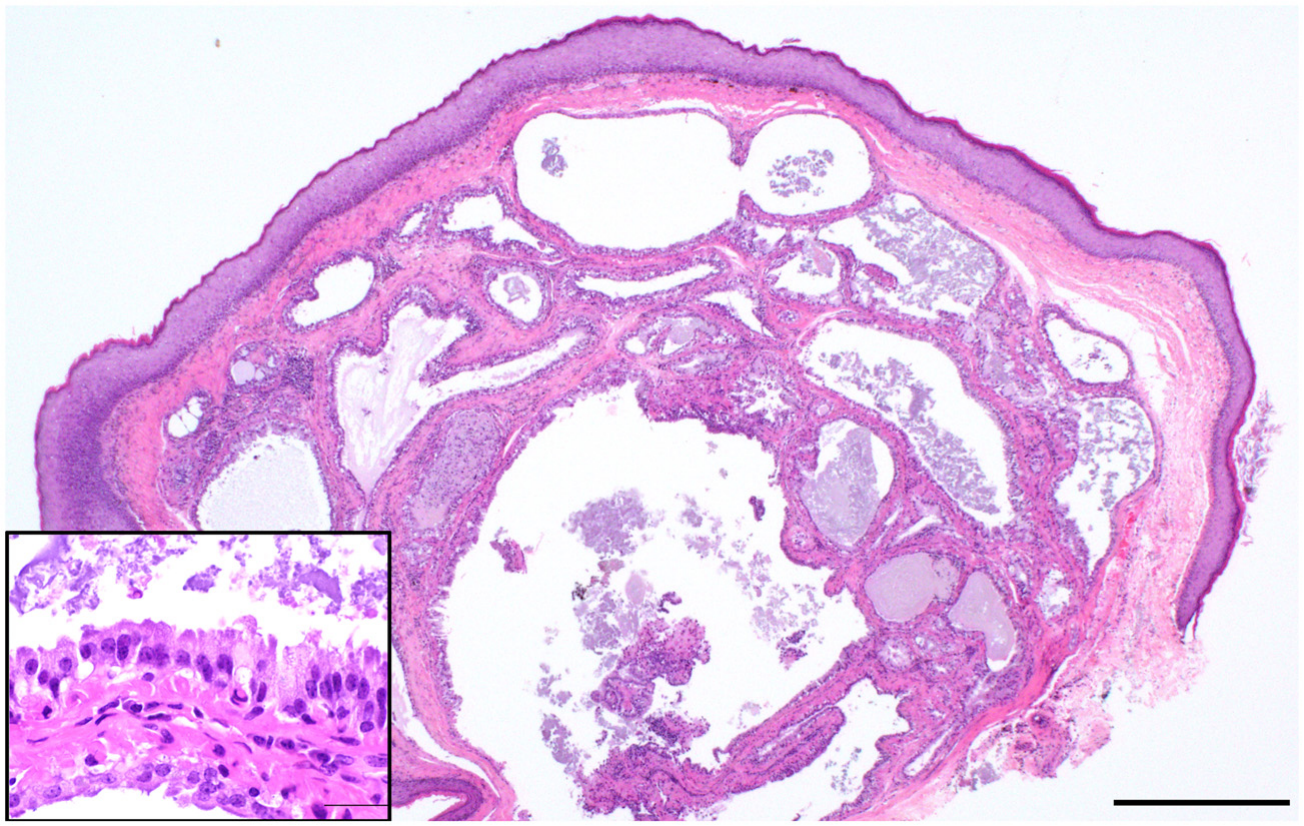
Photo of hematoxylin and eosin—stained histopathology slide of ceruminous gland cystadenocarcinoma in a Santa Catalina Island fox (main image scale bar = 1mm, inset = 50μm).

**Fig 6:**
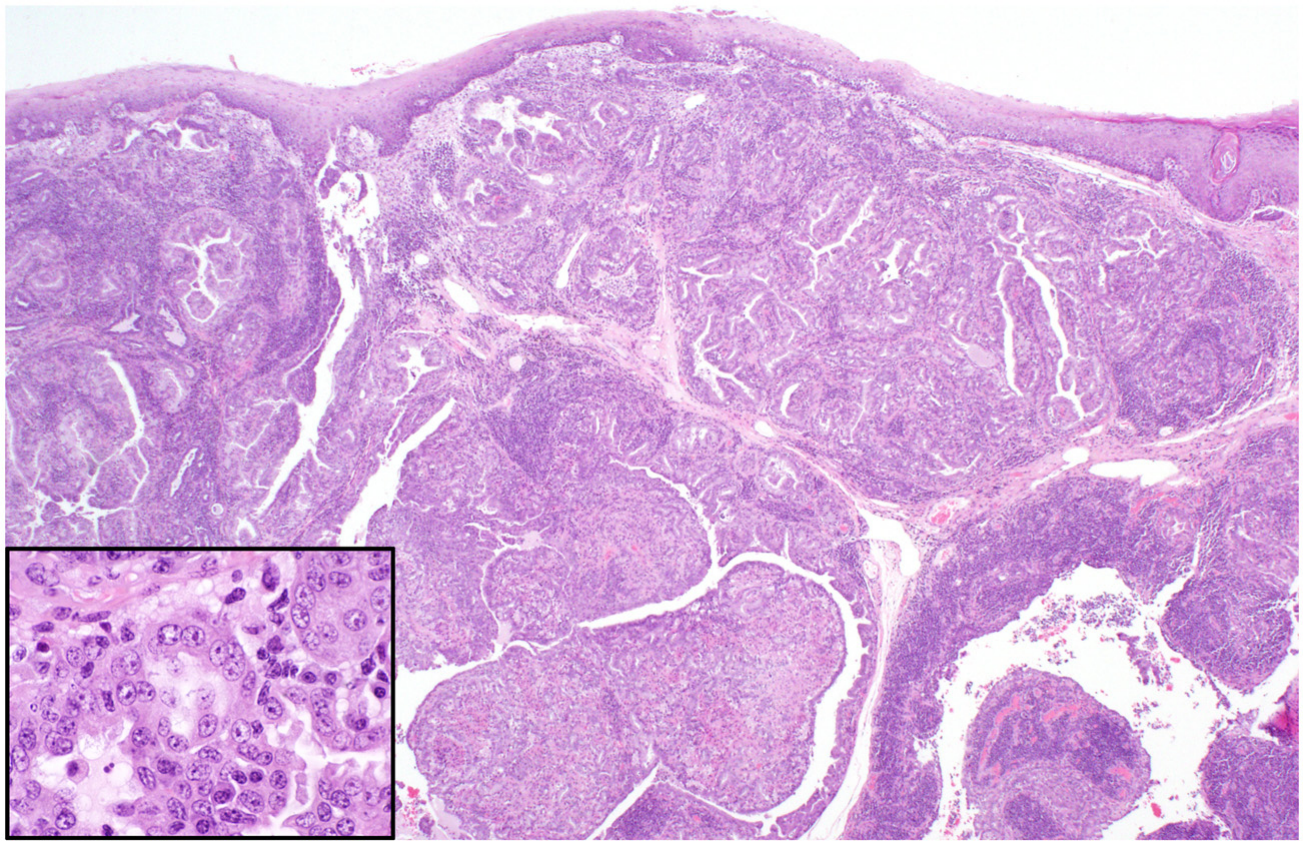
Photo of hematoxylin and eosin—stained histopathology slide of ceruminous gland solid adenocarcinoma in a Santa Catalina Island fox (magnification same as Fig 5, inset = 50μm).

**Fig 7:**
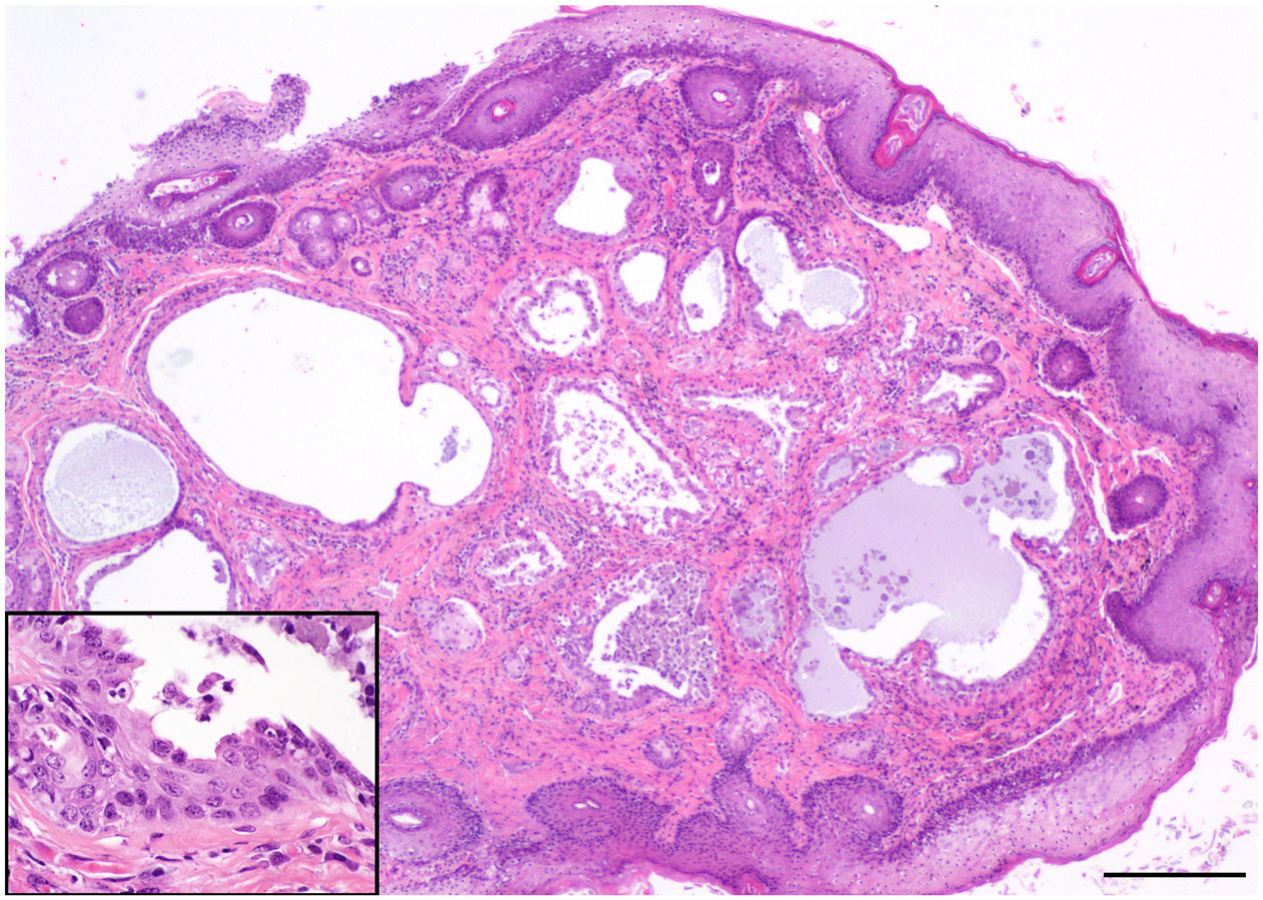
Photo of hematoxylin and eosin—stained histopathology slide of ceruminous gland dysplasia in a Santa Catalina Island fox (main image scale bar = 200 μm, inset = 50μm).

Histologic examination of necropsy and biopsy specimens from foxes with gross proliferative lesions revealed a spectrum of microscopic changes, both among cases as well as within individuals. Neoplastic proliferative lesions included adenomas, and cystic and solid carcinomas (cystadenocarcinoma; Fig 5; adenocarcinomas; Fig 6). Affected foxes had various degrees of chronic otitis externa accompanied by marked hyperplasia and hyperkeratosis of the epidermis and multifocal ulceration. Mites, identified as *Otodectes cynotis* (U.S. National Parasite Collection number 093186.00), were present in a majority of both live (98%) and deceased (58.5%) foxes with otitis. All tumor cases had varying degrees of ceruminous gland hyperplasia, some with papillary projections into the glandular lumen. In the areas of florid hyperplasia, the epithelium piled up into multiple disorganized layers and formed small nests and acini without breaching the basement membrane (dysplasia; Fig 7). In some cases, hyperplastic epithelium formed polypoid projections into the ear canal with a concurrent chronic inflammatory infiltrate (inflammatory polyps).

Adenocarcinomas consisted of neoplastic epithelial cells that were organized into tubules, acini, or sheets that breached the glandular basement membrane, invaded and replaced the deep dermis, and occasionally incited desmoplasia or invaded adjacent tissues. The neoplastic cells were cuboidal to flattened with a moderate amount of eosinophilic cytoplasm, a large oval nucleus, and one to multiple prominent nucleoli. Mitotic figures were regionally variable with as many as three per high power field (400x), and some tumor foci contained large central areas of necrosis. Adenomas were dermal and characterized as discrete, expansile, and non-invasive.

### Mites, microbes, viruses, and toxins

**Fig 8:**
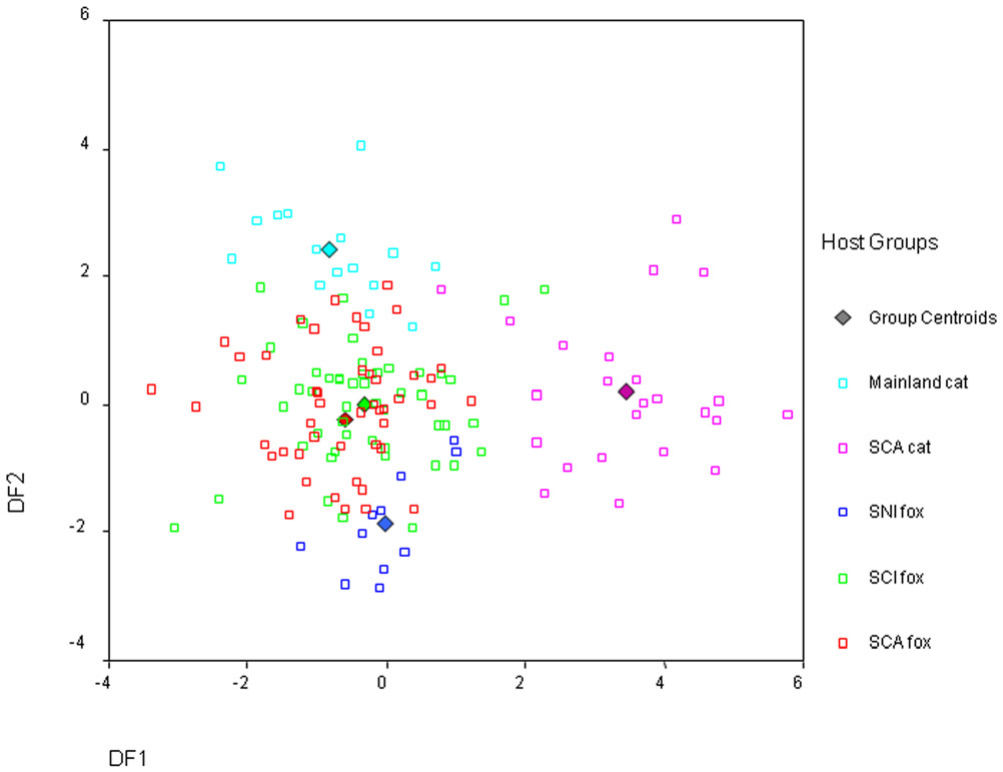
Scattergram developed from canonical discriminant analysis of 9 morphometric characters measured on *Otodectes cyanotis* mites from different hosts illustrating morphologic differentiation of mites present in island foxes from Santa Catalina (SCA), San Clemente (SCI) and San Nicolas Islands (SNI) and feral cats from SCA and the mainland. Discriminant functions 1 (DF1; body length and outer opisthosomal setae #3 length) and 2 (DF2; outer opisthosomal setae #3 length) cumulatively accounted for 87% of the variation in group measurement means observed.

The prevalence of ear mites was very high on all three islands—98.7% on SCA, and 100% on both SCI and SNI, and there was no difference in the intensity of infection (mite scores) among infected foxes on each island. Morphometric analyses provided evidence of phenotypic differentiation among mites from foxes on the three islands (Fig 8). Wilk’s lambda was significant for all four canonical discriminant functions, but the first two discriminant functions (DF1 and DF2) cumulatively accounted for 87% of the variation in the group measurement means observed. Based on standardized correlation coefficients, body length and outer opisthosomal setae #3 length were the most important morphologic characters to describe DF1 and outer opisthosomal setae #3 length was the most important morphologic character to describe DF2. Mites were most similar from foxes on the two islands closest to each other (SCA and SCI, Fig 1), while those from SNI clustered more distantly. Somewhat surprisingly, mites from cats on SCA and mainland California formed distinct clusters from each other and from all of the fox mites.

Foxes with tumors were no more likely to have bacteria or fungi that are regarded as potentially pathogenic in their ears than foxes without tumors. The most prevalent bacterial species cultured from ear canals across all the islands were *Staphylococcus* spp. and coliform bacteria. Pathogenic bacteria, including *Streptococcus* spp., *Pseudomonas* spp., and *Proteus* spp., were only rarely cultured, while *Malassezia* yeast infections were detected in all three populations. Herpesvirus and papillomavirus DNA were not detected in any of the frozen biopsy samples from the ear canals of 88 SCA foxes. As a result of the uniformly negative findings on the only island where tumors have been identified, virus testing was not conducted on samples from SCI and SNI foxes.

We detected low levels of PCB’s (≤ 1.4ppb) in the sera of two foxes (SNI = 1; SCA = 1). PCB and OC levels in adipose tissue were highest in SCI foxes, with four of five having quantifiable levels up to 1,500 ppb of multiple PCB and OC compounds. The lowest levels were in SCA foxes, where only two of five foxes had quantifiable levels of PCB’s (all levels ≤ 20 ppb) with no OC detected.

### Tumor prevalence and risk factor analysis

Tumors were highly prevalent among live and dead foxes from SCA from 2001–2008. In contrast, no tumors were detected in any of the live or dead foxes from SCI and SNI (Table 3). The prevalence of tumors among live mature foxes on SCA was 52.2% in 2007–2008, comparable to the estimated prevalence of 48.9% among foxes of all ages that died from 2001–2008 (Table 3). Carcinomas (34.8%) were twice as common as adenomas (17.7%) in mature foxes, and for foxes with tumors whose exact age was known (n = 27), the mean ages of diagnosis with carcinoma and adenoma were 5.8 and 4.9 years, respectively.

**Table 3:** The prevalence of tumors (ceruminous gland carcinomas and adenomas) among live (2007–2008) and dead (2001–2008) adult island foxes examined from Santa Catalina (SCA), San Clemente (SCI) and San Nicolas (SNI) islands in the Channel Islands of southern California, USA.

Study Group	N =	% Carcinoma^a^ (n)	% Adenoma (n)	% Tumors combined (n)
		[95% CI]	[95% CI]	[95% CI]
SCA live foxes	59	0	1.7% (1)	1.7% (1)
1–3 yrs old			[1.6–5.0%]	[1.6–5.0%]
SCA live foxes	46	34.8% (16)	17.4% (8)	52.2% (24)
≥ 4 yrs old		[20.3–47.7%]	[6.4–28.4%]	[37.8–66.7%]
SCA live foxes	105	15.2% (16)	8.6% (9)	23.8% (25)
total		[8.3–22.1%]	[3.2–14.0%]	[15.7–32.0%]
SCA dead foxes	47	40.4% (19)	8.5% (4)	48.9% (23)
		[26.4–54.4%]	[0.53–16.5%]	[34.6–63.2%]
SCI live foxes	59	0	0	0
SCI dead foxes	50	0	0	0
SNI live foxes	89	0	0	0
SNI dead foxes	50	0	0	0

^a^. Carcinoma category includes ceruminous gland adenocarcinomas and cystadenocarcinomas

**Fig 9:**
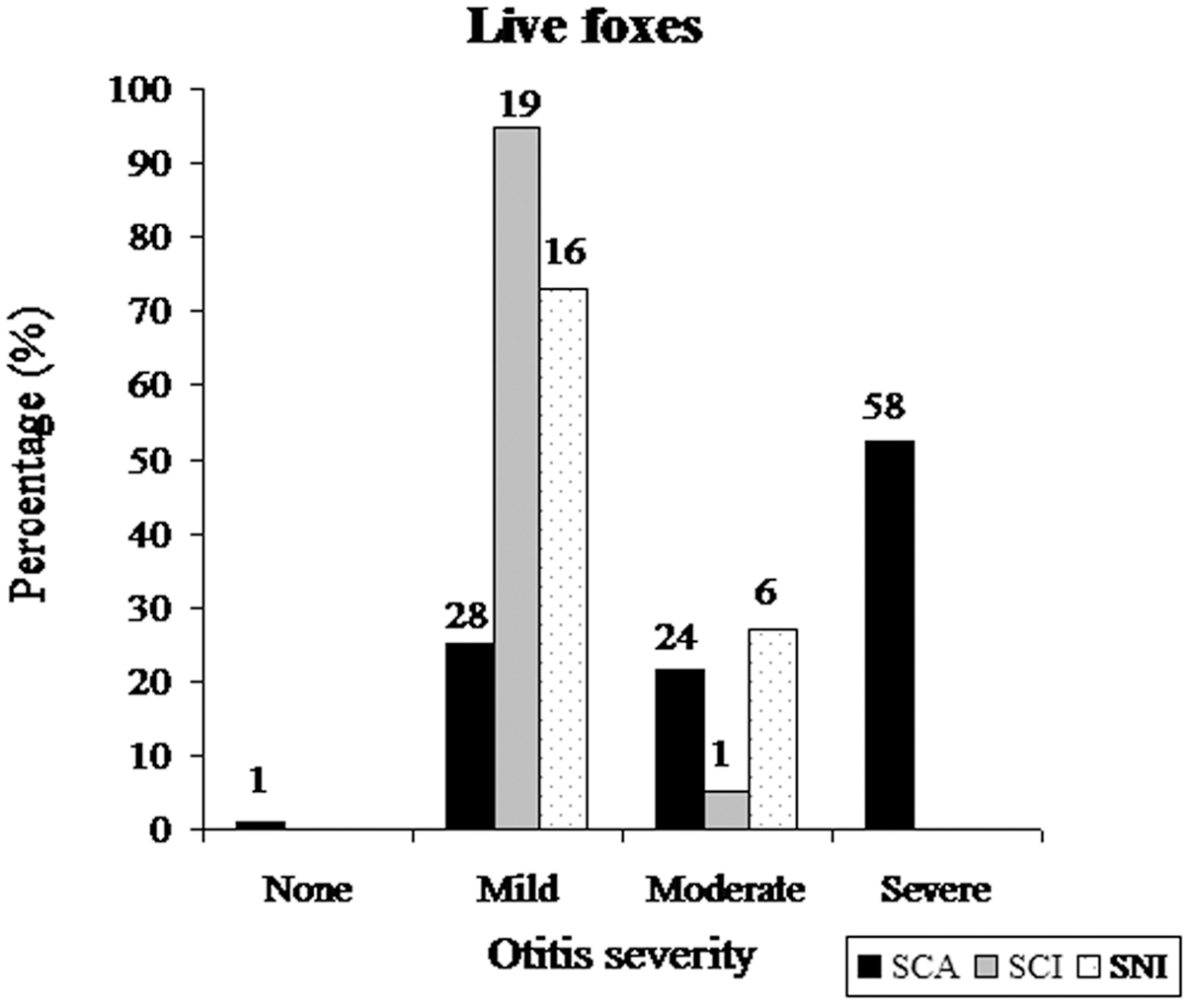
The frequency distribution of otitis severity scores in biopsy-sampled live adult foxes from SCA, SCI, and SNI. Number above bar equals sample size for each subgroup.

**Fig 10:**
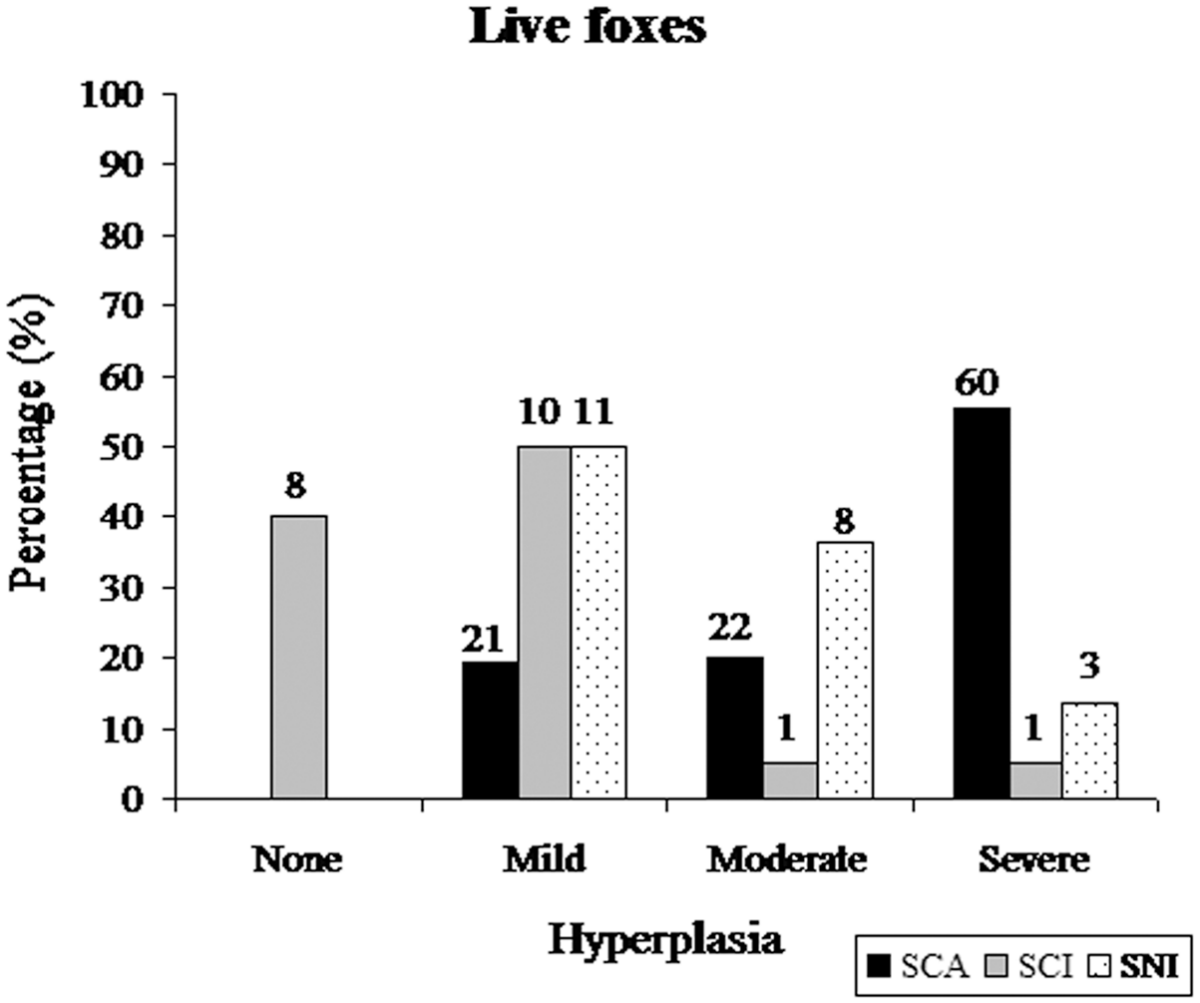
The frequency distribution of ceruminous gland hyperplasia scores in biopsy-sampled live adult foxes from SCA, SCI, and SNI. Number above bar equals sample size for each subgroup.

Mature live SCA foxes with tumors were 15 times more likely to have moderate to severe ceruminous gland hyperplasia (*X* ^2^ = 13.35, p < 0.001, 95% CI OR 3.0–70.6), and 3.6 times more likely to have ceruminous gland dysplasia (*X* ^2^ = 5.72, p = 0.017, 95% CI OR 1.4–9.5), than similarly aged SCA foxes without tumors. Though mature SCA foxes with tumors were not more likely to have moderate to severe otitis (*X* ^2^ = 2.49, p = 0.114) than those without tumors, otitis was more severe in live SCA foxes (KWANOVA *X* ^2^ (3 df) = 39.15, p <0.001) than in SCI or SNI foxes (Fig 9). A strong positive correlation between the severity of otitis and the severity of ceruminous gland hyperplasia (rho = 0.772, p<0.001) existed for all three islands. Ceruminous gland hyperplasia scores also differed among islands (KWANOVA *X* ^2^ (3 df) = 50.12, p < 0.001), with CGH being more prevalent and severe in SCA foxes than in SCI or SNI foxes (Fig 10). SCA foxes with tumors had significantly higher CGH scores (median = 3.0) than SCA foxes without tumors (median = 2.0), and foxes from SCI (median = 1.0) and SNI (median = 2.0). SCA foxes with tumors were 20 times more likely to have moderate to severe CGH (40/42; 95.2%) compared to foxes from all islands without tumors (54/108; 50%; chisq = 24.5, p < 0.001, 95% CI OR 4.6–86.9). Only 2 of 40 tumor cases had no or mild CGH.

Although the presence of mites was not a predictor of tumor occurrence, mite intensity scores were positively correlated with otitis severity scores (rho = 0.457, p < 0.001) and ceruminous gland hyperplasia severity (rho = 0.366, p = 0.002) in non-tumor foxes on SCA, but not in foxes on SCI or SNI. Correlations related to mite intensity scores were not significant for SCA foxes with tumors. Notably, increasing mite intensity scores were inversely associated with tumor diagnosis, thus animals without tumors were more likely to have higher mite intensity scores.

Findings from the multivariate logistic regression analysis on live-sampled SCA foxes indicated that older age, female sex, and increasing CGH severity score were all significantly associated with the presence of a tumor (Table 4). Age was the most significant risk factor with mature foxes (n = 76) being 13.6 times more likely to be diagnosed with a tumor than young foxes (P<0.001). In fact, only three tumor cases were diagnosed in the 35 young foxes sampled. Even when adjusted for age and sex, every incremental increase in CGH severity score resulted in a fox being 3.5 times more likely to be diagnosed with a tumor (P<0.001). Although not quite significant in the overall model, two of the three young animals diagnosed with tumors were female, thus including sex as a variable in the overall model improved fit.

**Table 4:** Final logistic regression model examining risk factors associated with the presence of a ceruminous gland tumor in live-sampled Santa Catalina Island foxes.

Risk Factor	SCA foxes with tumors		
	OR	95% CI	P value
Age > 4 years	13.61	3.37–54.89	<0.001
Sex (female)	2.83	0.98–8.19	0.054
CGH Score	3.49	1.74–6.98	<0.001
Mite Intensity Score	0.45	0.24–0.84	0.011

## Discussion

This study revealed that the endangered Santa Catalina Island fox had one of the highest rates of cancer ever found in a free-ranging wildlife population [18], comparable to that seen in Tasmanian devils (*Sarcophilus harrisii*) affected with devil facial tumor disease [28]. Prevalence estimates from the prospective study of live foxes (2007–2008) revealed ceruminous gland tumors in 52.2% of mature foxes (≥ 4 yrs of age), and a similar prevalence was found among deceased foxes (2001–2008). In contrast, no tumors were detected in foxes from San Nicolas Island or San Clemente Island, even though ear mites were highly prevalent on all three islands.

Pathologic examination showed that the fox tumors resemble ceruminous gland tumors commonly found in the domestic dog and cat [29–32]. Carcinomas and adenomas of the ceruminous gland are the most common neoplasms found in the external ear of dogs and cats [29]. Chronic inflammation, concurrent bacterial and ear mite infections, ceruminous gland hyperplasia, and excess cerumen production, are all considered predisposing factors for neoplastic transformation in these species [29]. More than two-thirds of the fox tumors were malignant cancers that invaded surrounding tissues, while less than a third were noninvasive adenomas contained within the ceruminous gland, the modified apocrine sweat gland that produces cerumen (ear wax). Some carcinomas were the proximate cause of death due to metastasis or sepsis secondary to tumor erosion of tissue and abscessation. Other tumors, both carcinomas and adenomas, also likely contributed to morbidity in some animals due to secondary bacterial and yeast infection. Because adenomas appear to be on the same development pathway as carcinomas, and some foxes diagnosed with adenomas later developed carcinomas in the same ear (unpublished data), we also consider the high prevalence of adenomas to be significant to this population.

We propose a conceptual model for the formation of tumors in the ceruminous glands of foxes on SCA, similar to that proposed for dogs [29]. Ear mite infections are acquired soon after birth when pups are in intimate contact with their mite-infected mother. Mite infections persist throughout life if left untreated, resulting in chronic, ongoing inflammation (otitis). Chronic inflammation leads to local tissue damage and repair, resulting in ceruminous gland hyperplasia, ectasia and dysplasia. As these pathologic changes persist and increase in severity over time, proliferative lesions develop, and benign adenomas and malignant carcinomas arise and become more prevalent as foxes age. Moriarty et al. [10] demonstrated that mite removal on SCA significantly reduced otitis and hyperplasia, thus establishing a strong association between mites and pathologic changes in the ear, thus providing support for this model.

A key question is “Why do ceruminous gland tumors only occur in SCA foxes, and not in foxes on the other two islands where ear mites are also common?” This study demonstrated that the consequences of mite infections are very different among SCA foxes than SCI or SNI foxes, with the ensuing inflammatory response and associated hyperplasia being much more severe among mite-infected live foxes on SCA. Furthermore, foxes with tumors on SCA were much more likely to have ceruminous gland hyperplasia than foxes without tumors, suggesting that tumors are a consequence of progressive and persistent inflammatory processes that are more severe in SCA foxes. Though we did not find positive correlations between mite intensity and otitis or CGH in live foxes with tumors, we speculate that large amounts of liquid pus and exudate that were common in foxes with tumors may have resulted in mite “washout” from the ear canal.

We hypothesized that the severity of disease on SCA could be due to differences among mites on the different islands, or differences in exposure to toxins or other pathogenic microbes. Although we detected morphologic differences among mite populations on the different islands and mainland, these appear to be the result of limited gene flow and isolation by distance. We did not identify a mite phenotype unique to SCA, thus finding no support for the hypothesis that mites unique to SCA are driving the development of tumors on this island. Furthermore, there were no associations between tumor development and bacteria, viruses, or toxins, suggesting these were not factors in the tumor development process.

Another possibility for the severity of lesions seen on SCA is that island foxes on SCA are unable to develop an adequate immune response against ear mite infection. However, Moriarty et al. [10] showed that SCA foxes do mount a strong IgG antibody response to mites, and that the level of antibody declines with reduction in mite intensity. Although foxes can produce antibodies to mite infections, there may well be other immunologic or genetic factors that influence the outcome of infection. The foxes on SCA are a separate subspecies from populations on other islands, and the genetic differences that exist between island fox populations have just begun to be characterized [14,33,34]. There may well be a genetic basis for increased susceptibility of SCA foxes to tumor formation, especially given that certain dog breeds such as cocker spaniels are predisposed to otitis externa and ceruminous gland tumors [35]. Furthermore, recent studies have shown that there can be a genetic basis for susceptibility to tumors in wild carnivores, including urogenital carcinomas in California sea lions (*Zalophus californianus*) [36], and devil facial tumor disease in Tasmanian devils [37]. We consider this one of the most important areas for further inquiry, and genetic studies have been initiated to identify genetic differences among foxes from SCA, SNI, and SCI that may influence their susceptibility to tumors.

The occurrence of cancer in wildlife is a growing concern given its potential to impact population persistence and even ecosystem function [38]. In one of the most striking and well-studied examples, devil facial tumor disease caused a 95% population decline among Tasmanian devils within a few years of initial detection [27]. The loss of this apex predator then set off a series of trophic cascades, potentially impacting native mesocarnivores [39,40]. Options for managing this disease are quite limited—there is no known treatment for these infectious tumors, and culling is unlikely to stop its spread [41].

We do not know how ceruminous gland tumors will affect the long-term survival of the SCA fox population, or if the high prevalence of tumors has impacts at the ecosystem level. However, the development of the conceptual model described in this paper gives us the basis for generating and testing hypotheses, including management actions that may reduce morbidity and mortality. Since persistent mite infections appear to play a key role in tumor development, a proportion of the foxes on SCA have been treated annually with acaricides since 2012. This study, and that of Moriarty et al [10], will provide the essential baseline data for comparison with future studies to assess whether or not treatment reduces the prevalence of mites and tumors, and enhances age-specific survivorship and longevity.

## Dedication

This manuscript is dedicated to the memory of Dr. Linda Munson, 1948–2010, who was one of the initiators of this study and helped guide and contribute to it throughout. She was committed to investigating cancer and other diseases in island foxes as well as many other wildlife populations. This study could not have been completed without her. She was a valued teacher, mentor, colleague, and friend to all the authors, and innumerable others in the veterinary, wildlife conservation, and pathology communities. She remains an inspiration to all who are concerned with the persistence of healthy animal populations throughout the world.
